# Supplementary material for: Delayed Access to Medical Care and Psychological Distress among Chinese Immigrants in Canada during the Pandemic
Source: Healthcare (Basel). 2024 Aug 16;12(16):1639. doi: 10.3390/healthcare12161639 (PMC11353734; doi:10.3390/healthcare12161639)
Supplement: Supplementary file 1 [file healthcare-12-01639-s001.zip › healthcare-3086904-supplementary.pdf]

Supplemental material – S1. Sample characteristics and distributions of variables in the study

Table S1.1. Sample characteristics (N=888)

Variables	Frequency	Percent
Age		
Under 35	58	6.53
35-44	116	13.06
45-54	291	32.77
55-64	229	25.79
65 or above	164	18.47
Missing	30	3.38
Gender		
Men	287	32.32
Women	554	62.39
Missing	47	5.29
Birthplace		
Mainland China	810	91.22
Other	47	5.29
Missing	31	3.49
Language		
Mandarin	790	88.96
Other	63	7.09
Missing	35	3.94
Province		
Ontario	759	85.47
Other	95	10.70
Missing	34	3.83
Length in Canada		
Under 6 years	91	10.25
6 to less than 15 years	269	30.29
At least 15 years	495	55.74
Missing	33	3.72
Marital status		
Married/Common law	735	82.77
Other	120	13.51
Missing	33	3.72
Living status		
With family	758	85.36
Other	83	9.35
Missing	47	5.29
Highest educational status		
Under university	201	22.64
University	371	41.78
Master/PhD	284	31.98
Missing	32	3.60
Employment status		
Employed	351	39.53
Self-employed	160	18.02

Variables	Frequency	Percent
Unemployed	116	13.06
Other	229	25.79
Missing	32	3.60
Work in healthcare		
Yes	71	8.00
No	766	86.26
Missing	51	5.74
Work requiring public contact		
Yes	147	16.55
No	691	77.82
Missing	50	5.63
Financial status		
Dissatisfied	197	22.18
Neutral	344	38.74
Satisfied	299	33.67
Missing	48	5.41
Health status		
Poor	41	4.62
Fair	336	37.84
Good	452	50.90
Missing	59	6.64
Covid-19 infectious history		
Yes	4	0.45
No	884	99.55
Fear of Covid-19		
Yes	568	63.96
No	214	24.10
Missing	106	11.94
Discriminate		
Perceived/experienced	320	36.04
No	506	56.98
Missing	62	6.98
Delayed medical care access		
Yes	634	71.40
Non-response about delay	254	28.60
CPDI		
Normal (<28)	419	47.18
Mild to severe (≥28)	327	36.82
Missing	142	15.99
Social Loneliness Score		
Observed	730	
Mean ± SD	17.45 ± 3.19	
Missing	158	

Abbreviations: CPDI=COVID-19 Peritraumatic Distress Index; SD=Standard deviation

Supplemental material – S2. Selection models and sensitivity analysis

We used proc hpgenselect with selection method = lasso (choose=aic stop=aic) to select the best variables for the final model. From the output for 20 selected models, we decided to exclude working requiring public contact variable in the model based on the frequency of selection and coefficient strength. **Table S2.1** presents variables selected across 20 imputed datasets. Eight variables: (a) age, (b) length in Canada, (c) highest educational status, (d) financial status, (e) fear of Covid-19, (f) delay, (g) social loneliness score, and (h) current health status chosen in most models are appropriate variables for the final model. Working requiring public contact were chosen in five models, which might indicate its lower consistency across imputed datasets. Regarding coefficient strength, the coefficient magnitude of working requiring public contact is significantly lower than that of other variables, suggesting that this variable had a lower impact on the final model compared to other variables. Moreover, we examined the final models with and without working requiring public contact (**Table S2.2**). There are no differences between two models. We evaluated the goodness-of-fits of imputed datasets using Hosmer & Lemeshow Goodness-of-Fit Test and Area Under the Receiver Operating Characteristics Curve (ROC) (**Table S2.3**). p-values obtained from the Hosmer & Lemeshow Goodness-of-Fit Test were greater than 0.05, and ROC values ranged from 0.7 to 0.8, indicating that the models were good-of-fitness.

Table S2.1. Model selection from logistic LASSO regression with imputed data

**Fit statistics**	**Imputed 1**	**Imputed 2**	**Imputed 3**	**Imputed 4**	**Imputed 5**	**Imputed 6**	**Imputed 7**	**Imputed 8**	**Imputed 9**	**Imputed 10**
AIC	812.84	812.42	812.01	809.15	806.12	810.14	**805.60**	812.83	809.77	813.03
BIC	854.37	858.57	853.54	855.29	847.65	851.68	**847.13**	854.37	851.30	854.56
**Variables**					**Parameter estimates**					
Age	0.1006	0.1016	0.1047	0.1085	0.1114	0.1080	0.1080	0.1016	0.1068	0.1030
Length in Canada	0.0810	0.0852	0.0788	0.0892	0.0863	0.0848	0.0892	0.0795	0.0885	0.0931
Highest educational status	0.0453	0.4723	0.0485	0.0520	0.0547	0.0507	0.0540	0.0444	0.0471	0.0499
Financial status	0.1247	0.1240	0.1369	0.1206	0.1159	0.1166	0.1183	0.1284	0.1214	0.1154
Fear of Covid-19	0.6783	0.6715	0.6433	0.6749	0.6714	0.6648	0.6828	0.6618	0.6646	0.6795
Delayed medical care access	0.2122	0.2191	0.1936	0.2206	0.2271	0.2253	0.2228	0.2235	0.2243	0.2262
Social Loneliness Score	-0.3257	- 0.3280	-0.3256	-0.3328	-0.3334	-0.3295	-0.3331	-0.3255	-0.3291	-0.3217
Work requiring public contact	-	0.0027	-	0.0037	-	-	-	-	-	-
Current health status	0.0144	0.0090	0.0155	0.0121	0.0117	0.0131	0.0052	0.0077	0.0046	0.0122

Fit statistics	Imputed 11	Imputed 12	Imputed 13	Imputed 14	Imputed 15	Imputed 16	Imputed 17	Imputed 18	Imputed 19	Imputed 20
AIC	807.07	808.00	805.86	810.61	808.65	809.62	808.80	807.97	808.78	808.63
BIC	848.60	849.53	847.40	852.14	850.10	855.77	854.95	849.50	843.70	854.78
**Variables**	**Parameter estimates**									
Age	0.1075	0.1105	0.1151	0.1071	0.1086	0.1059	0.1104	0.1062	0.1058	0.1112
Length in Canada	0.0924	0.0852	0.0936	0.0852	0.0818	0.0896	0.0896	0.0830	0.0855	0.0815
Highest educational status	0.0535	0.0535	0.0572	0.0522	0.0431	0.0449	0.0537	0.0513	0.0445	0.0542
Financial status	0.1194	0.1196	0.1234	0.1231	0.1323	0.1212	0.1166	0.1297	0.1222	0.1279
Fear of Covid-19	0.6898	0.6695	0.6862	0.6679	0.6713	0.6735	0.6794	0.6695	0.6613	0.6758
Delayed medical care access	0.2274	0.2308	0.2303	0.2264	0.2176	0.2201	0.2217	0.2204	0.2208	0.2240
Social Loneliness Score	-0.3276	-0.3324	-0.3302	-0.3291	-0.3292	-0.3308	-0.3326	-0.3306	-0.3317	-0.3328
Work requiring public contact	-	-	-	-	-	0.0022	0.0030	-	-	0.0014
Current health status	0.0091	0.0131	0.0101	0.0066	0.0060	0.0111	0.0075	0.0028	-	0.0052

Abbreviations: AIC=Akaike information criterion; BIC=Bayesian information criterion

Table S2.2. Multivariate logistic regression

Variables	Completed cases analysis			Imputed cases analysis[a]			Imputed cases analysis[b]		
	OR	95% CI	p-value	OR	95% CI	p-value	OR	95% CI	p-value
Age									
Under 35	1.946	0.858 – 4.411	0.3016	1.317	0.734 – 2.363	0.3551	1.301	0.726 - 2.332	0.3770
35-44	1.534	0.802 – 2.934	0.7602	1.111	0.723 – 1.706	0.6307	1.108	0.720 - 1.704	0.6418
45-54	1.510	0.897 – 2.540	0.7458	1.065	0.780 – 1.455	0.6899	1.067	0.781 - 1.458	0.6823
55-64	1.343	0.771 – 2.339	0.7201	0.952	0.666 – 1.361	0.7867	0.948	0.663 - 1.355	0.7678
65 or above	ref			ref			ref		
Length in Canada									
Under 6 years	1.339	0.771 – 2.339	0.6733	1.108	0.759 – 1.616	0.5961	1.110	0.761 - 1.621	0.5875
6 to less than 15 years	1.403	0.924 – 2.129	0.3770	1.120	0.844 – 1.486	0.4307	1.119	0.843 - 1.485	0.4372
At least 15 years	ref			ref			ref		
Highest educational status									
Under university	1.303	0.793 – 2.143	0.2299	1.170	0.880 – 1.556	0.2797	1.158	0.870 - 1.542	0.3133
University	0.999	0.661 – 1.510	0.4690	0.943	0.743 – 1.196	0.6263	0.945	0.744 - 1.199	0.6414
Master/PhD	ref			ref			ref		
Financial status									
Dissatisfied	1.314	0.803 – 2.150	0.3736	1.133	0.852 – 1.507	0.3910	1.136	0.854 - 1.511	0.3809
Neutral	1.168	0.776 – 1.758	0.9176	1.004	0.792 – 1.272	0.9757	1.000	0.789 - 1.267	1.0000
Satisfied	ref			ref			ref		
Fear of Covid-19									
Yes	2.617	1.694 – 4.041	<.0001	1.604	1.293 – 1.989	<.0001	1.605	1.294 - 1.991	<.0001
No	ref			ref			ref		
Delayed medical care access									
Yes	1.900	1.184 – 3.048	0.0078	1.362	1.078 – 1.720	0.0095	1.357	1.075 - 1.715	0.0103
Non-response about delay	ref			ref			ref		
Social Loneliness Score	1.384	1.291 – 1.483	<.0001	1.408	1.314 – 1.508	<.0001	1.406	1.313 - 1.507	<.0001
Work requiring public contact							ref		
Yes	1.245	0.782 – 1.982	0.3563	-	-	-	1.109	0.879 - 1.400	0.3816
No	ref			-	-	-	ref		
Current health status									
Poor	-	-	-	1.529	0.815 – 2.869	0.1860	1.505	0.803 - 2.820	0.2022
Fair	-	-	-	0.782	0.544 – 1.124	0.1838	0.789	0.549 - 1.134	0.2008
Good	-	-	-	ref			ref		

[a]. The final multivariate logistic regression model contain age, length in Canada, highest educational status, financial status, fear of Covid-19, delay, social loneliness score, and current health status.

[b]. The final multivariate logistic regression model contain age, length in Canada, highest educational status, financial status, fear of Covid-19, delay, social loneliness score, working requiring public contact, and current health status.

Abbreviations: CPDI=COVID-19 Peritraumatic Distress Index; 95% CI=95% Confidence interval; OR=Odd ratios

Table S2.3. Check goodness-of-fit and ROC across unimputed and imputed models

	Unimputed dataset	Imputed 1	Imputed 2	Imputed 3	Imputed 4	Imputed 5	Imputed 6	Imputed 7	Imputed 8	Imputed 9	Imputed 10
Hosmer & Lemeshow Goodness-of-Fit Test[a]	0.5027	0.8316	0.8096	0.8010	0.6006	0.3361	0.4058	0.3859	0.6962	0.4555	0.8546
ROC curve[b]	0.7960	0.8061	0.8072	0.8067	0.8087	0.8090	0.8070	0.8096	0.8057	0.8067	0.8058

	Imputed 11	Imputed 12	Imputed 13	Imputed 14	Imputed 15	Imputed 16	Imputed 17	Imputed 18	Imputed 19	Imputed 20
Hosmer & Lemeshow Goodness-of-Fit Test[a]	0.4830	0.4766	0.3833	0.6913	0.2882	0.7156	0.4404	0.5662	0.5688	0.3627
ROC curve[b]	0.8088	0.8080	0.8090	0.8065	0.8080	0.8089	0.8089	0.8081	0.8075	0.8089

[a]. p-value > 0.05 indicates that the model has a goodness-of-fit. (David et al. (2013), p 165)
[b]. Area Under the Receiver Operating Characteristics Curve indicates the ability of the fitted model to discriminate between individuals with and without outcome of interest (David et al. (2013), p.174-178)

- $0.7 \leq ROC < 0.8$: indicates an acceptable discrimination.
- $0.8 \leq ROC < 0.9$: indicates an excellent discrimination.

References

Hosmer D.W., Lemeshow S., & Sturdivant R.X. (2013, April). Applied Logistic Regression. John Wiley & Sons. ISBN: 978-0-470-58247-3
